# Impressions

**Published:** 1868-07

**Authors:** S. Clippinger


### IMPRESSIONS.

BY S. CLIPPINGER.

By request I give you my manner of taking impressions. I adopted it in 1861, and, as yet, have failed to find any other way that will give me such general satisfaction. I find it better adapted for difficult cases than any thing I have tried. Others may not succeed so well with it as with some other methods of taking impressions, simply from the fact, perhaps, that they do not get at it just right, for we differ many times on modes of operating; while one is perfectly attached to a mode or material, another, of equally good standing in the profession will condemn the mode or the material, or both. Some say with cylinders only can a good gold filling be inserted. Others, again, select sponge or crystal gold, this is more from the manner of manipulation than the material. While one is successful with cylinders and fails with the adhesive preparations of gold, the other fails with the former, and attains success with the latter, so we find what is a success with one may not be with another, simply for the want of proper application on the part of the operator; not that the principle is not correct or the material the best calculated to accomplish the desired result. So when we fail let us be slow to condemn, for the material fails for the want of proper manipulation, and the principal for the want of skill in demonstration. In the first place I select an impression cup to suit the case in hand, then use beeswax, put it in warm water and let it soften to the proper consistency or temperature, then fill the cup selected; take the impression in the ordinary way of taking a wax impression. After it is removed from the mouth (if the weather is warm I apply cold water to harden the wax,) then trim it to form just such a cup as I want, (which is easier done than told,) if for an impression of the upper jaw, the back part will require trimming off to the proper length, and the rim outside of the alveolar ridge will want more or less trimming down. Should the ridge be very prominent and sunk in under the lip and cheeks so that the wax impression on the outer rim overhangs, cut or trim it so as to remove that overhanging portion. If for a lower set trim the edges off so as to give just such a cup as is needed, or should it be for a partial upper set, say the four biscuspids, trim the back end as before, and anterior to the six front teeth cut the wax away half way down the crowns, and the same with the molars on each side. Should any of the remaining teeth be so shaped or lean in any way so as to cause the impression to hang, then cut the wax away so as to leave a space of an eighth of an inch between such teeth and the wax on the inside. But if for a partial lower set, three teeth back on each side, the eight front teeth remaining, then cut the wax away in front of the teeth as before, and on the inside of the front teeth trim so as to leave a space of an eighth of an inch over that portion where I want the plate, only trimming the edges of the wax back of the teeth.

Now, the object of trimming the wax impression is to give just such a cup for the plaster impression as is needed; one that will give a plaster impression of uniform thickness; one that can not spring up in the center of the arch, (if an upper impression,) and can neither contract or expand, all of which I have to contend with; the impression is taken in plaster with an ordinary cup. After the wax cup (for such it is,) is trimmed, then mix the calcined plaster to the consistency, say of thick cream, stir it till the bubbles are gone, then fill the wax cup with it, being careful not to get in too much plaster, (for if careful there will be but very little surplus plaster in the patient's mouth,) then press the cup to its place in the mouth, after the plaster has set, which can, by raising or lowering the lip, (as the case may be,) readily be seen. If an upper impression, then start it first by raising the lip and cheek on one side and press the cup down at the heel so as to let the air under, or between the impression and mouth, when it will readily let go; though, sometimes, it will hold very firm, so that I have to work for some little time to get it started. If the mouth is so shaped that it can not draw, then, where the wax has been cut away, the plaster will be more or less broken. In that case I am careful to get the pieces and replace them, which I am able to do with more certainty on the wax than with an ordinary cup, from the fact that it is more firm, consequently the impression will be less broken, and will be sharper, owing to the cup fitting the mouth so snugly; and having trimmed off the over-hanging portion, the plaster will be correspondingly thick at those points, so that it does not crumble as it otherwise would. I proceed the same way with all the other cases. If the partial upper set, the plaster outside of the remaining natural teeth will break off as the cup is removed, and that is just what I want, and if any on the inside of the teeth break off I replace it as before, when all is right, the partial lower piece works the same. Some may say, or think at least, that they could succeed just as well with the common cup and plaster. All I have to say is that I can not, though I have tried plaster alone and all the different preparations of wax, but as often have returned to the wax cup trimmed to suit my wants, and as often have been compelled to give in to it, as being superior to all other modes of taking impressions. I find the wax cup an advantage in many respects other than those I have mentioned. For instance, in the mouth of an aged person where the lower jaw has wasted away, the cheeks fleshy, glands full, so that I have the patient raise the tongue to get them from under the cup, and I have to press the cheek out, or I have an impression of the fleshy parts of the mouth instead of the jaw, but with my wax cup I seldom fail, not so with plaster alone. Again, where the upper jaw is fleshy over a portion of the surface of the hard palate, and the rest hard or firm, I find it of great advantage, the wax cup presses the soft portion firmly, so that when taken by the coating of the wax cup with plaster it is nearer the pressure of the plate than any other way that I have attempted.

